# Improved Separation of Odor Responses in Granule Cells of the Olfactory Bulb During Odor Discrimination Learning

**DOI:** 10.3389/fncel.2020.579349

**Published:** 2020-10-09

**Authors:** Dejuan Wang, Yang Chen, Yiling Chen, Xiaowen Li, Penglai Liu, Zhaoyang Yin, Anan Li

**Affiliations:** Jiangsu Key Laboratory of Brain Disease and Bioinformation, Research Center for Biochemistry and Molecular Biology, Xuzhou Medical University, Xuzhou, China

**Keywords:** granule cells, fiber photometry, odor representation, go/no go, go/go

## Abstract

In the olfactory bulb, olfactory information is translated into ensemble representations by mitral/tufted cells, and these representations change dynamically in a context-dependent manner. In particular, odor representations in mitral/tufted cells display pattern separation during odor discrimination learning. Although granule cells provide major inhibitory input to mitral/tufted cells and play an important role in pattern separation and olfactory learning, the dynamics of odor responses in granule cells during odor discrimination learning remain largely unknown. Here, we studied odor responses in granule cells of the olfactory bulb using fiber photometry recordings in awake behaving mice. We found that odors evoked reliable, excitatory responses in the granule cell population. Intriguingly, during odor discrimination learning, odor responses in granule cells exhibited improved separation and contained information about odor value. In conclusion, we show that granule cells in the olfactory bulb display learning-related plasticity, suggesting that they may mediate pattern separation in mitral/tufted cells.

## Introduction

Interpreting the dynamic environment precisely to facilitate appropriate behavior is crucial for animal survival. To accomplish this complex task, sensory systems in the brain encode dynamic information in the activity of neuronal ensembles. Such representations in the sensory system form an important constituent of information processing in the brain (Andermann et al., 2010; Komiyama et al., 2010; Harvey et al., 2012; Huber et al., 2012; Kato et al., 2015). As the first processing center in the olfactory system, the olfactory bulb (OB) plays an important role in odor detection and discrimination (Wilson et al., 2017; Chong and Rinberg, 2018; Li et al., 2020). Odor representation in the OB is highly dynamic and is modulated by various types of olfactory experience (Kass et al., 2013; Abraham et al., 2014; Kass et al., 2016; Hu et al., 2020b; Li et al., 2020). Previous studies have established that odor representations in OB output neurons (mitral/tufted cells, M/T cells) display improved pattern separation during active odor discrimination learning (Nunez-Parra et al., 2014; Yamada et al., 2017; Wang et al., 2019) when mice have learned to discriminate two odors, the representation of those odors in M/T cells becomes more divergent. This process is thought to convey information about odor value and improve odor discrimination learning (Nunez-Parra et al., 2014; Li et al., 2015). Nevertheless, it remains unknown how pattern separation in M/T cells arises.

Pattern separation could arise from the native OB network during odor discrimination learning. In the OB, olfactory information relayed by the olfactory sensory neurons (OSNs) is transmitted to M/T cells, which in turn send their axons to higher brain areas (Uchida et al., 2014; Vaaga and Westbrook, 2016). Studies have shown a lack of odor experience-dependent plasticity and learning-related pattern separation in the OSN input (Kato et al., 2012; Chu et al., 2017), indicating that pattern separation in M/T cells is not inherited from changes in the OSN inputs to the OB. Rather, it may be due to plasticity downstream of the OSN inputs, e.g., within the synaptic interactions with the interneurons in the OB. The activity of M/T cells is extensively modified by dynamic interactions with GABAergic and dopaminergic interneurons within the OB (Burton, 2017). These interneurons form both dendro-dendritic reciprocal synapses and axo-dendritic synapses with M/T cells and mediate lateral and recurrent inhibition onto M/T cells (Margrie et al., 2001; Aungst et al., 2003; Hu et al., 2017, 2020a), which play a major role in transforming odor representations (Nusser et al., 2001; McGann, 2013). Previous studies have shown that odor enrichment can induce response changes in the inhibitory interneurons of the OB (Mandairon et al., 2008) and that GABAergic inhibition onto M/T cells is crucial for pattern separation and odor discrimination (Abraham et al., 2010; Godde et al., 2016).

Granule cells (GCs) are a major class of GABAergic interneurons in the OB and provide feedback inhibition to M/T cells through reciprocal dendrodendritic synapses (Isaacson and Strowbridge, 1998; Abraham et al., 2010). Importantly, GCs not only regulate OB output to other brain regions but also mediate top-down modulation of sensory processing in the OB (Boyd et al., 2012; Nunez-Parra et al., 2013). Previous studies have shown that GCs exhibit strong odor responses and impose a sparse and temporally dynamic structure on the ensemble activity of M/T cells (Kato et al., 2012; Cazakoff et al., 2014). Exciting or inhibiting GCs in the OB bidirectionally alters pattern separation in the M/T cells and olfactory discrimination (Gschwend et al., 2015; Nunes and Kuner, 2015). The important role of GCs in pattern separation raises the possibility that GCs may mediate pattern separation in M/T cells. However, little is known about how odor responses in OB GCs change during odor discrimination learning.

In this study, we used fiber photometry to characterize the activity of the GCs population in awake behaving mice engaged in an odor discrimination task. We found that odor responses in GCs were excitatory and reliable. When mice were proficient in the discrimination task, odor responses in GCs exhibited improved separation, suggesting that odor responses of GCs in the OB display context-dependent plasticity and contain information about odor value.

## Materials and Methods

### Animals

C57BL/6J male mice aged 10–14 weeks old were used for fiber photometry recordings. Before surgery, mice were housed under a 12/12 h light/dark cycle and housed in groups. After surgery, they were housed individually for at least 2 weeks for recovery before further experiments. All mice were given *ad libitum* access to chow and water except during the behavioral sessions. During the behavioral sessions, mice were weighed daily and received sufficient water to maintain > 80% of their pre-water-restriction weight. Animal care and use conformed to protocols submitted to and approved by the Xuzhou Medical University Institutional Animal Care and Use Committee.

### Virus Injection and Fiber Implant

We used the genetically encoded Ca^2+^ indicator GCaMP6s to monitor the activity of neurons. EGFP-expressing animals were used as controls for comparison with GCaMP6s-expressing animals. AAVs used in this study, including AAV-VGAT1-Cre (AAV2/9, 5.26 × e+12 vg/mL), AAV-DIO-GCaMP6s (AAV2/9, 5.33 × e+12 vg/mL), and AAV-DIO-EGFP (AAV2/9, 4.98 × e+12 vg/mL) were purchased from BrainVTA (Wuhan, China). For targeted viral delivery, mice were fixed in a stereotactic frame (RWD, Shenzhen, China) under pentobarbital sodium anesthesia (i.p. 90 mg/kg). A small craniotomy was made and a calibrated pulled-glass pipette (Sutter Instrument) was lowered to the OB (coordinates 4.28 mm from lambda, 1.00 mm from the midline, and 1.20 mm ventral to lambda). A total volume of 300 nl of virus (AAV-VGAT1-Cre and either AAV-DIO-GCaMP6s or AAV-DIO-EGFP, in a 1:2 mixture) was injected with a microsyringe pump (Stoelting Quintessential Injector) at a rate of 40 nl/min. The injection pipette was left in place for ten additional minutes before being withdrawn slowly.

For optical manipulation, following virus injection mice were implanted with custom-built fiber connectors [0.37 numerical aperture (NA), 200 μm diameter; Newdoon]. The tip of the fiber was lowered to the injection site in the OB. The optical fiber was fixed in place with dental acrylic and a custom-made aluminum head-plate was attached to the skull to enable head-fixation. After surgery, mice were housed individually for at least 2 weeks to allow sufficient time for transgene expression and animal recovery. At the end of the behavioral analyses, we sacrificed the subject mice, performed standard histology, and confirmed the efficiency of both AAV infection and fiber placement.

### Fiber Photometry Recording

Fiber photometry was performed using a previously described system (Zhou et al., 2017; Sun et al., 2019; Wang et al., 2019, 2020; Wu et al., 2020). To record fluorescent signals, the beam from a 488 nm laser (OBIS 488LS, Coherent) was reflected by a dichroic mirror (MD498, Thorlabs), focused by an objective lens (10×, NA: 0.3; Olympus), and then coupled to an optical commutator (Doric Lenses). An optical fiber (200 mm o.d., NA: 0.37, 1.5 m long) coupled the light between the commutator and the implanted optical fiber. GCaMP6s fluorescence was collected by the same fiber and objective, then bandpass-filtered (MF525–39, Thorlabs) and detected by a photomultiplier tube (R3896, Hamamatsu). An amplifier (C7319, Hamamatsu) converted the photomultiplier tube current output to a voltage signal, which was further filtered through a low-pass filter (35 Hz cut-off; Brownlee, 440). The analog voltage signals were digitized at 500 Hz and recorded by fiber photometry software (Thinkerbiotech, Nanjing, China) for the duration of each behavioral session.

### Odor Delivery

Odors were dissolved in mineral oil at 1% (v/v) dilution. Similar to our previous studies (Wang et al., 2019; Liu et al., 2020), eight odors that always induce frequent responses were used during passive exposure: isoamyl acetate, 2-heptanone, phenyl acetate, benzaldehyde, dimethylbutyric acid, n-heptane acid, n-pentanol, and 2-pentanone (Sinopharm Chemical Reagent). In the go/no go task, only the first two pairs of odors (isoamyl acetate versus 2-heptanone and phenyl acetate versus benzaldehyde) were used. As in our previous studies (Wang et al., 2019; Liu et al., 2020), isoamyl acetate and phenyl acetate were defined as S+ odors, and 2-heptanone and benzaldehyde as S– odors. Odors were presented by an odor delivery system (Thinkerbiotech, Nanjing, China). A stream of charcoal-filtered air flowed over the oil and was then diluted to 1/20 by an olfactometer. Odor presentation was synchronously controlled by the data acquisition system via a solenoid valve driven by a digital-to-analog converter. Air or odorized air was delivered at a constant rate of 1 l/min to eliminate the effect of airflow. The duration of each odor presentation was 2 s and the inter-trial interval was 30 s.

### Overview of Training and Behavioral Tasks

After recovering from surgery, mice were head-fixed with two horizontal bars but were able to maneuver on an air-supported free-floating Styrofoam ball (Figure 4A). During passive exposure, the eight odorants were delivered randomly, with 15 trials for each odorant. Before starting the behavioral tasks (a go/go task and a go/no go task), mice were water restricted and their weight was maintained at 80–85% of their initial weight. During the behavioral task, mice performed daily sessions that lasted 200 trials, or until the mouse disengaged, whichever came first. On each trial, one of two odorants was pseudorandomly delivered (maximum of two trials in a row with the same odorant). Each trial consisted of a 2 s odorant delivery period, followed by a 0.5 s answer period, during which the mouse could choose whether or not to lick a lickport (Figure 4B). Mice were trained to perform a go/go task during which a water reward was delivered from the lick-port when either of the odorants was delivered on a trial and the mouse responded by licking the lickport during the answer period. A 15 s inter-trial interval followed the answer period, and there was no punishment on error trials.

Next, the mice were trained to perform a go/no go task in which they were required to discriminate the reinforced odor (S+) from the unreinforced odor (S–) to receive the water reward. In this task, mice learned to lick the lickport when an S+ was presented and to not lick the lickport when an S– was presented. Thus, if an S+ was presented and the mouse responded with licking (Hit), a water reward was delivered through the lickport; if they failed to lick in response to the S+ (Miss) the water reward was not delivered. If an S– was presented, water was never delivered, regardless of the mouse’s actions [licking in response to an S– was classed as a false alarm (FA); not licking in response to an S– was classed as a correct rejection (CR); see Figure 4C]. Hits and CRs were classed as correct responses, whereas Misses and FAs were classed as incorrect responses. The performance was evaluated in blocks of 20 trials, with 10 S+ and 10 S– trials presented at random. The percentage correct value for each block represents the percentage of trials in which the odors were correctly discriminated and associated with the appropriate behavioral action. Each session included 6–10 blocks of 20 trials. Calcium signals were recorded simultaneously throughout the behavioral tasks.

### Immunohistochemistry

For verification of viral expression, frozen brain sections were prepared. The mice were deeply anesthetized with pentobarbital sodium (i.p. 90 mg/kg) and perfused intracardially with 0.9% saline, followed by 4% paraformaldehyde (PFA) in PBS (0.1 M, pH 7.4). The brains were subsequently removed and postfixed in 4% PFA at 4°C overnight. After cryoprotection with 30% (w/v) sucrose, brain tissue was then embedded in OCT compound and coronal sections (30 μm) were cut on a cryostat (Leica CM1860). Sections were incubated with blocking solution (5% normal goat serum, 0.3% Triton X-100 in PBS) and incubated for 2 h at room temperature. Primary antibodies (anti-GAD67, 1:250, MAB5406, Millipore) were diluted in blocking solution and applied overnight at 4°C. Sections were washed three times with PBS and incubated with fluorescent secondary antibodies for 2 h at room temperature. After washing three times in PBS, slides were incubated with DAPI for nuclear staining and coverslipped with a 50% glycerol mounting medium. Images were obtained by confocal scanning microscopy (Zeiss, LSM710) and were processed via ZEN 2011 (Zeiss).

### Statistical Analysis

#### Behavioral Performance

For the go/go and go/no go tasks, the performance in each block was calculated as follows: (number of Hit trials + number of CR trials)/total number of trials, including all Hit, Miss, CR, and FA trials.

#### Analysis of Fiber Photometry Data

Data were exported as MATLAB .mat files and segmented according to the onset of odor stimulation on individual trials. We derived the values of fluorescence change (ΔF/F) by calculating (F – F_0_)/F_0_, where F_0_ is the baseline fluorescent signal averaged over a 5 s long control time window, which preceded the onset of odor stimulation. Averaged ΔF/F values for 5 s from the onset of odor delivery are presented as heat maps or trial-averaged plots. In the go/go and go/no go tasks, the first 30 trials in the first session were classified as “naïve” trials, and the last 30 trials in the last session were classified as “proficient” trials.

#### ROC Analysis

Receiver operating characteristic (ROC) analysis was used to assess the classification of the responses evoked by odor pairs. ROCs were estimated using the roc function from the MATLAB exchange. The area under the ROC (auROC) is a nonparametric measure of the discriminability of two distributions. We used auROC to assess the classification of two odors within an odor pair. The area under the ROC curve was defined as ranging from 0.5 to 1.0. A value of 0.5 indicates completely overlapping distributions, whereas a value of 1.0 indicates perfect discriminability.

#### Calculation of Differences in ΔF/F

We used the difference in ΔF/F to assess the extent of the divergence in the responses to two odors within an odor pair. The responses evoked by the two odors were defined as Res A and Res B, respectively. The difference in ΔF/F was calculated as follows: ABS (Res A—Res B)/Res A, where ABS represents the absolute value and Res A and Res B represent the responses evoked by odor A and odor B, respectively.

All statistical analyses were performed with MATLAB or Prism. The Shapiro-Wilk test was used to assess the normality of the data. We used the Friedman test, Mann-Whitney test, Wilcoxon’s sign rank test, and paired *t*-test; all tests were two-sided. All data in the present study are presented as the mean ± SEM.

## Results

### Excitatory Responses to Odors in GCs Recorded With Fiber Photometry

**FIGURE 1:**
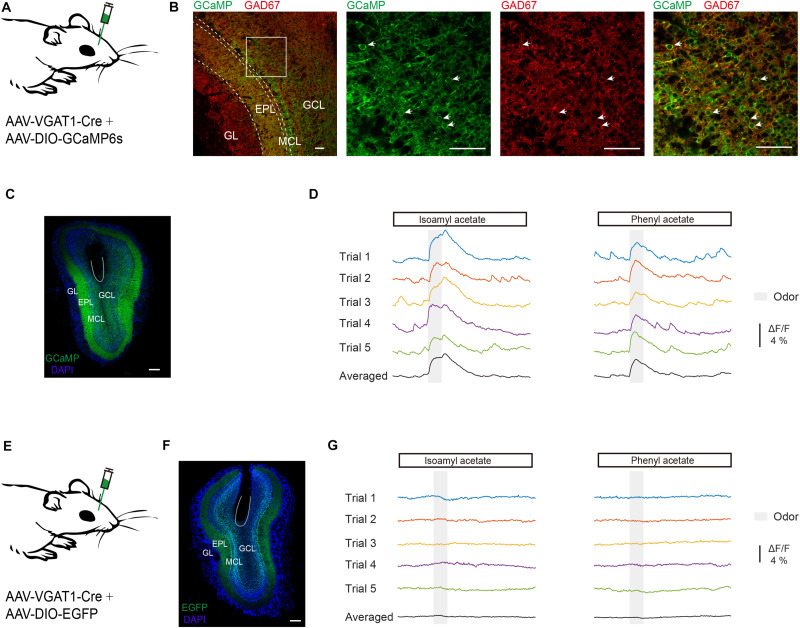
Odor-evoked responses are recorded in GCs of the OB. **(A)** Diagram of virus injection. To record the odor-evoked calcium responses of granule cells, AAV-VGAT1-Cre and AAV-DIO-GCaMP6s were injected into the OB of C57BL/6J mice. **(B)** Expression pattern in the OB following virus injection. Note the dense labeling in the granule cell layer and the external plexiform layer. Expression of GCaMP6s in the granule cell layer co-localizes with immunolabeling of GAD67. GL, Glomerular layer; EPL, external plexiform layer; MCL, mitral cell layer; GCL, granule cell layer. Scale bar = 50 μm. **(C)** Representative viral transduction (GCaMP6s) and fiber location. Expression of GCaMP6s in the OB. Within the OB, GCaMP6s is expressed in the granule cells. Scale bar = 200 μm. **(D)** Typical traces and trial-averaged traces of the calcium responses evoked by isoamyl acetate and phenyl acetate. **(E)** Diagram of virus injection. AAV-VGAT1-Cre and AAV-DIO-EGFP were injected into the OB of C57BL/6J mice. **(F)** Representative viral transduction (EGFP) and fiber location. Scale bar = 200 mm. **(G)** Odors evoked no response in a control mouse.

First, we recorded odor-evoked response profiles in GCs. It is reported that VGAT is expressed in all GABAergic neurons (Vong et al., 2011) and the VGAT-Cre animal line has been used to study the activity of GCs in the OB (Fukunaga et al., 2014; Wienisch and Murthy, 2016). Neuronal activity of GCs was monitored with the genetically encoded Ca^2+^ indicator GCaMP6s in awake head-fixed mice using fiber photometry. GCaMP6s expression was genetically restricted to GCs by injecting a composite virus solution (AAV-VGAT1-Cre and AAV-DIO-GCaMP6s) into the granule cell layer of the OB in C57BL/6J mice (Figure 1A). Three weeks after viral injection there was an extensive expression of GCaMP6s in the granule cell layer and the external plexiform layer where the dendrites of granule cells are distributed (Figures 1B,C). GCaMP6s fluorescence in the granule cell layer represents expression in GCs while GCaMP6s fluorescence in the mitral cell layer and the external plexiform layer largely reflects dendrites of GCs. GCaMP6s expression was restricted to GCs as shown by colocalization with immunolabeling of GAD67 in the granule cell layer (Figure 1B). We observed an increase of Ca^2+^ levels during and after odor application in the GC population of the OB (Figure 1D). C57 BL/6J mice injected with a mixture of AAV-VGAT1-Cre and AAV-DIO-EGFP served as controls (Figures 1E,F): no calcium signal was detected in these mice (Figure 1G, *n* = 4 mice). Thus, this method allowed us to selectively record odor-evoked responses from GCs located beneath the optical fiber.

**FIGURE 2:**
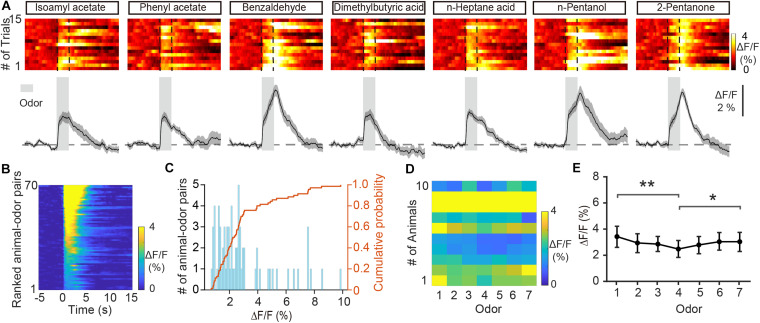
Odor-evoked responses in GCs of the OB are excitatory. **(A)** Trial-to-trial pseudocolored heat maps (top) and trial-averaged traces (bottom) of the calcium responses evoked by different odors. **(B)** Heat maps of ΔF/F averaged across all trials and further averaged across odorants for each mouse, ranked by mean ΔF/F. **(C)** Histograms and cumulative probability of ΔF/F. **(D,E)** Pseudo-color heat-map of ΔF/F **(D)** and averaged ΔF/F **(E)** evoked by different odors. [Friedman test: χ^2^(6, 54) = 18.8, *P* = 0.0046, odor 1 versus odor 4, *P* = 0.00106, odor 4 versus odor 7, *P* = 0.0428]. *P < 0.05, **P < 0.01.

Previous fiber photometry and two-photon Ca^2+^ imaging studies have shown that M/T cells display both excitatory and inhibitory responses to odors (Yamada et al., 2017; Wang et al., 2019). Unlike M/T cells, GCs showed only increases in Ca^2+^ levels in response to odor delivery (Figures 2A–C, from 0.71 to 9.86%, average: 2.94 ± 0.252%, *n* = 70 animal-odor pairs from ten mice). To investigate how GCs respond to different odorants, we compared the odor responses between the different odorants. We found that the averaged ΔF/F was significantly different for different odorants (Figures 2D,E, Friedman test, *P* = 0.00460; odor 1 versus odor 4, *P* = 0.00106; odor 4 versus odor 7, *P* = 0.0428). Therefore, different odors induce different excitatory responses in the GC population of the OB.

**FIGURE 3:**
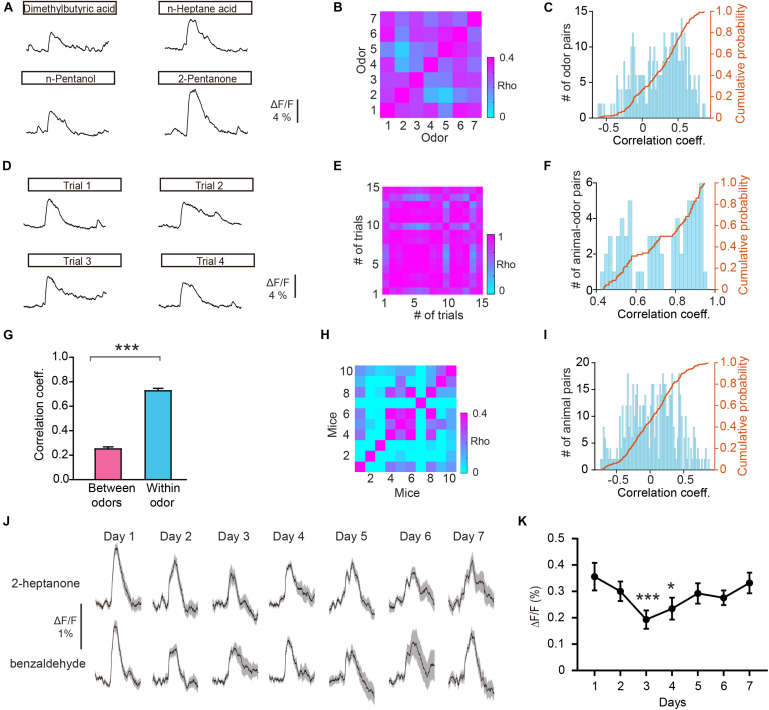
Odor responses differ between odors. **(A)** Odor responses evoked by four odorants in an individual mouse. **(B,C)** Heat map **(B)**, histograms **(C)**, and cumulative probability **(C)** of the correlation coefficients for the ΔF/F induced by different odorants. **(D)** Odor responses evoked by the same odorant (n-Pentanol) in different trials in an individual mouse. **(E,F)** Heat map **(E)**, histograms **(F)**, and cumulative probability **(F)** of the correlation coefficients for the ΔF/F from different trials with the same odorant. **(G)** Comparison of the correlation coefficients for the “between odors” and “within odor” conditions (Mann-Whitney test, U = 2706, *P* < 0.0001). **(H,I)** Heat map **(H)**, histograms **(I)**, and cumulative probability **(I)** of the correlation coefficients for the ΔF/F induced by the same odor in different animals. **(J)** Representative GC odor responses over 7 days of passive exposure. **(K)** Averaged ΔF/F over days. [Friedman test: χ^2^(6, 138) = 26.7, *P* = 0.000164, odor 1 versus odor 4, *P* = 5.96 × 10^– 5^, odor 4 versus odor 7, *P* = 0.0281]. *P < 0.05, ***P < 0.001.

To further investigate whether odorants are differentially represented in the GC population, we computed the Pearson’s correlation coefficient. This measures the similarity between pairs of population vectors constructed from the responses to pairs of different odors. Figure 3A shows the odor responses evoked by four different odorants in an individual mouse. We found that the correlation between responses to different odors (between odors) was low (Figures 3B,C, *r* = 0.252 ± 0.0595). To exclude the possibility that recorded differences were caused by the instability of fiber photometry recording, we calculated the correlation coefficient for different trials within individual odorants (within odor) (Figures 3D–F, *r* = 0.726 ± 0.0197). We found that the within-odor correlation coefficients were much larger than the between-odors correlation coefficients (Figure 3G, Mann–Whitney test, *P* < 0.0001). These results suggest that odor responses in GCs is different between odors while remaining stable within the same odor. Next, we investigated whether the responses to the same odor were distinct in different animals. We found that the correlation between responses to the same odor in different animals was low (Figures 3H,I, *r* = 0.0365 ± 0.0141). This suggests that the different animals display distinct odor responses recorded with fiber photometry.

Previous studies using two-photon calcium imaging have shown that daily odor experience could induce a gradual weakening of mitral cell activity (Kato et al., 2012; Yamada et al., 2017). We examined whether GC odor responses display such experience-dependent change in response strength over days. We repeated the passive odor application for 7 consecutive days. We observed a slight weakening of odor responses in GCs at days 3 and 4 (Figures 3J,K, Friedman test, *P* = 0.000164; day 1 versus day 3, *P* = 5.96 × 10^–5^; day 1 versus day 4, *P* = 0.0281). This result suggests that similar to that in mitral cells, GC responses also display an experience-dependent change in response strength.

### Improved Separation of Odor Responses in GCs After Odor Discrimination Learning

**FIGURE 4:**
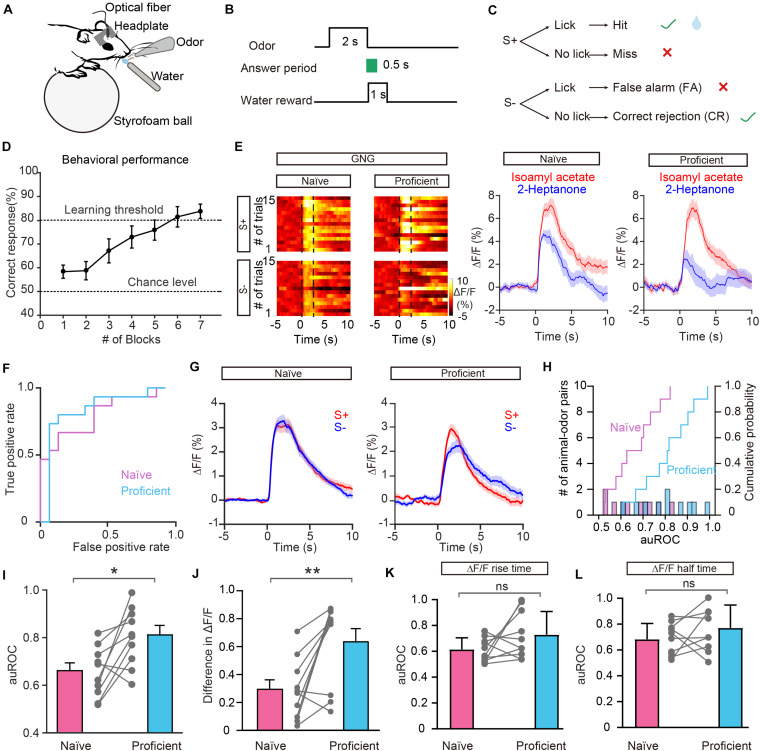
Odor responses in GCs show improved separation in the go/no go (GNG) task. **(A)** Diagram of the experimental set-up. **(B)** Timeline for a single trial. Each trial consisted of a 2 s odorant delivery period, followed by a 0.5 s answer period during which the mouse could choose whether or not to lick a lickport. A water reward was delivered if the stimulus was rewarded (S+) and the mouse licked during the answer period. **(C)** Schematic of behavioral paradigm. If an S+ was presented and the mouse responded with licking, a water reward was delivered. If an S– was delivered, no water reward was delivered regardless of the mouse’s actions. **(D)** Odor discrimination performance during the last session in the go/no go task. The mean percentage correct responses are plotted for each block of 20 trials. The chance level and learning threshold are indicated by dashed lines. **(E,F)** Heat maps **(E)**, peri-event plots **(E)**, and ROC graph **(F)** of Ca^2+^ signals from an individual mouse induced by S+ and S– in the naïve stage and in the proficient stage during the go/no go task. **(G)** Averaged traces of odor responses across all animal-odor pairs (*n* = 10 animal-odor pairs from nine mice). **(H)** Histograms and cumulative probability of auROCs in the naïve stage and the proficient stage. **(I,J)** auROCs **(I)** and difference in ΔF/F **(J)** are larger in the proficient stage than in the naïve stage. [**I:** paired *t*-test, *t*_(__9)_ = 3.15, *P* = 0.0118; **J:** Wilcoxon’s sign rank test, *W* = –49.0, *P* = 0.00980.] **(K,L)** Comparison of the auROC values of ΔF/F rise time **(K)** and half time **(L)** between the naïve stage and proficient stage. [**(K)** paired *t*-test, *t*_(__9)_ = 1.68, *P* = 0.127; **(L)** paired *t*-test, *t*_(__9)_ = 1.32, *P* = 0.221]. *P < 0.05, **P < 0.01.

In addition to encoding information about odor identity, M/T cells display enhanced separation of odor representations when animals are learning an odor discrimination task (Nunez-Parra et al., 2014; Yamada et al., 2017; Wang et al., 2019), and GCs play an important role in that separation (Gschwend et al., 2015). We therefore asked whether odor responses in the GC population show increased separation when mice have learned to discriminate a pair of odors. To address this question, mice were trained on a go/no go discrimination task (Figure 4A). First, mice underwent a pre-training period to learn a go/go paradigm, in which they were presented with two odorants, both of which were paired with the water reward. Mice were trained to lick the lick-port in response to odorants to obtain the water reward. After reaching the learning threshold in the go/go task (80% correct), mice were then trained to perform a go/no go discrimination task in which they eventually learned to lick in response to the rewarded odorant (S+) to obtain a water reward while refraining from licking to the unrewarded odorant (S–), which was not paired with a water reward (Figures 4B,C). Behavioral performance was assessed by calculating the percentage of correct responses to S+ and S– in each 20-trial block with mice completing 6–10 blocks per session. Performance gradually improved over the week-long training period. On the last day of go/no go training, performance improved from near chance levels (50% correct) in Block 1 to well above the learning threshold (80% correct) in Block 6 (Figure 4D).

Figure 4E shows odor-evoked responses in GCs from an individual mouse performing the go/no go task. The traces are sorted into trials where the mouse was learning to differentiate the odorants (left, first 30 trials of the first session, naïve) and trials where mice were proficient in discriminating the odorants (right, last 30 trials of the last session, proficient). The difference in odor responses evoked by S+ and S– increased once the mouse had learned to discriminate the odorants (Figure 4E). Analysis of ΔF/F evoked by S+ and S– showed that the auROC values were larger during the proficient period than during the naïve period (Figure 4F). The separation of odor responses evoked by S+ and S– was observed consistently for other animal-odor pairs. The averaged ΔF/F values during the naïve and proficient periods for all animal-odor pairs (*n* = 10 animal-odor pairs from nine mice) are shown in Figure 4G. Both the auROC values and the difference in ΔF/F increased when mice became proficient in the go/no go task (Figures 4H–J, auROC: paired *t*-test, *P* = 0.0118; difference in ΔF/F: Wilcoxon’s sign rank test, *P* = 0.00980). These data demonstrate that odor responses in GCs display enhanced separation after odor discrimination learning. We also analyzed the ΔF/F rise time and half time during the go/no go task and found that there is no significant difference in the auROC values of ΔF/F rise time or half time between the naïve period and proficient period (Figure 4K, paired *t*-test, *P* = 0.127; Figure 4L, paired *t*-test, *P* = 0.221).

### Lack of Improved Separation of Odor Responses in GCs During the Go/Go Task

**FIGURE 5:**
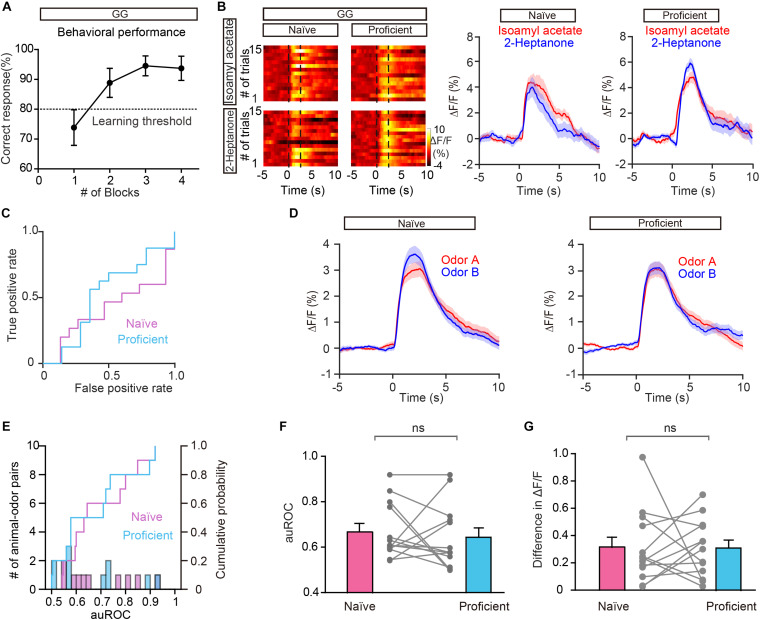
Odor responses in GCs do not show separation during the go/go (GG) task. **(A)** Percentage of correctly answered trials in the last session of the go/go task. **(B,C)** Heat map **(B)**, peri-event plot **(B)**, and ROC graph **(C)** of Ca^2+^ signals from an individual mouse in the naïve stage and in the proficient stage of the go/go task. **(D)** Averaged traces of odor responses across all animal-odor pairs (*n* = 13 animal-odor pairs from ten mice). Odor A and odor B represent the two rewarded odors delivered during the go/go task. **(E)** Histograms and cumulative probability of auROCs in the naïve stage and the proficient stage. **(F,G)** auROCs **(F)** and the difference in ΔF/F **(G)** are not significantly different between the naïve stage and the proficient stage. [**(F)** Wilcoxon’s sign rank test, *W* = 14.0, *P* = 0.622; **(G)** Wilcoxon’s sign rank test, *W* = -15.0, *P* = 0.636].

To exclude the possibility that the improved separation in the go/no go task is due to general behavioral state (such as thirst) differences between the naïve period and the proficient period, we compared the odor responses during these two periods in the go/go task where mice also received water and became satiated. Figure 5A shows the behavioral performance of all mice performing the go/go task (*n* = 13 animal-odor pairs from ten mice). Odor responses showed no increase in separation during the proficient period (Figures 5B–D). Further analysis indicated that neither the auROC values nor the difference in ΔF/F was significantly different between naïve trials and proficient trials during the go/go task (Figures 5E–G, auROC, Wilcoxon’s sign rank test, *P* = 0.622, difference in ΔF/F, Wilcoxon’s sign rank test, *P* = 0.636). Therefore, the improved separation of odor responses in GCs during the go/no go task is established by learning-related plasticity as opposed to behavioral states.

### GCs Encode Odorant Value

**FIGURE 6:**
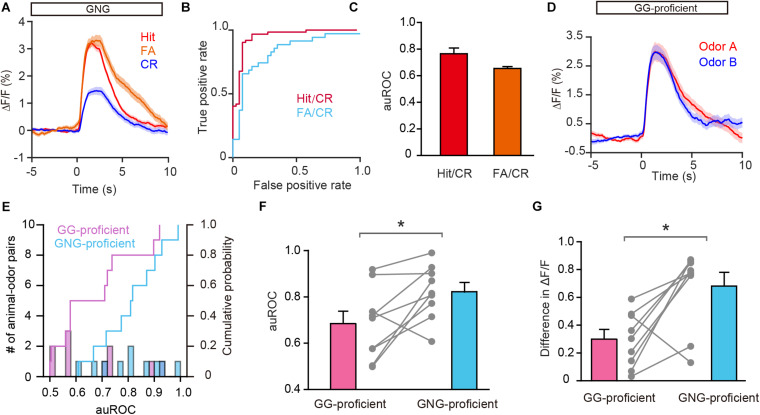
Odor responses in GCs reflect odor value as opposed to odorant identity. **(A)** Averaged traces for Hit, FA, and CR trials during the go/no go (GNG) task across all animal-odor pairs. **(B)** ROC analysis for Hit/CR and FA/CR from an individual mouse. **(C)** The auROCs for Hit/CR trials and FA/CR trials across all mice performing the go/no go task. **(D)** Averaged traces of odor responses during the go/go (GG) task (*n* = 9 animal-odor pairs from eight mice), odor A and odor B represent the rewarded odors delivered during the go/go task. **(E)** Histograms and cumulative probability of auROCs in the proficient stage for the go/go task and the go/no go task. **(F,G)** The auROCs **(F)** and difference in ΔF/F **(G)** in the proficient stage for the go/go task and the go/no go task. [**F:** paired *t*-test, *t*_(9)_ = 3.01, *P* = 0.0146; **G:** Wilcoxon’s sign rank test, *W* = -37.0, *P* = 0.0273]. *P < 0.05.

Previous studies have shown that both odor-induced oscillations in the OB and odor responses in M/T cells differ between FA and CR trials (Ramirez-Gordillo et al., 2018; Wang et al., 2019), which are incorrect and correct responses to the same odorant (S–), respectively. Using the methods described above, we analyzed GC activity during the Hit, FA, and CR trials in the last session of the go/no go task. Figure 6A shows that activity on the CR trials was well separated from activity on both the Hit and FA trials. We then performed an ROC analysis using FA/CR and Hit/CR trials. The auROC was significantly different from zero (the diagonal) for both FA/CR and Hit/CR [Hit/CR, *t*_(__9)_ = 6.13, *P* = 0.0002; FA/CR, *t*_(__9)_ = 11.5, *P* < 0.0001, one-sample *t*-test, Figures 6B,C]. Thus, the calcium signal can distinguish correct (CR) from incorrect (FA) responses relatively well, even though both are responses to the same odorant (S–). This indicates that odor responses in GCs reflect odor value as opposed to odor identity.

To investigate this further, we asked whether GCs display improved separation in the go/go task, in which both odorants delivered are rewarded. To address this, we analyzed data from mice performing the go/go task and then the go/no go task with the same odor pair. As shown in Figure 6D, odor responses evoked by the two odors during the proficient period in the go/go task did not display separation. Both the auROC and the difference in ΔF/F were significantly greater in the go/no go task than in the go/go task (Figures 6E,F, paired *t*-test, *P* = 0.0146; Figure 6G, Wilcoxon’s sign rank test, *P* = 0.0273, *n* = 9 animal-odor pairs from eight mice). In other words, no separation was observed when odorant valences were the same (in the go/go task) but significant separation was observed when odorant valences were different (in the go/no go task). Therefore, similar to studies showing that odor value is encoded by M/T cell activity (Nunez-Parra et al., 2014; Wang et al., 2019), odor responses in GCs also contain information about odor value.

## Discussion

Studies have shown that the neural activity of M/T cells displays improved pattern separation during active learning and conveys information about odor value (Doucette and Restrepo, 2008; Doucette et al., 2011; Gschwend et al., 2015; Wang et al., 2019). Here, we explored the change of odor responses of OB granule cells during odor discrimination learning. Using fiber photometry, we characterized the basic odor response properties of GCs and, then tracked the long-term changes in population odor responses: we discovered that GC responses to pairs of odors display improved separation during a go/no go task. The responses of the same odor differed on FA and CR trials and improved separation was not observed during the go/go task, suggesting that GC activity contains information about odor value. Therefore, odor responses in GCs display learning-related plasticity and may mediate the pattern separation observed in M/T cells.

Although GCs are the major class of GABAergic interneurons in the OB, direct *in vivo* measurement of GC activity in awake animals has been limited to only a few studies demonstrating that GC activity is modulated by brain state and respiration (Kato et al., 2012; Cazakoff et al., 2014; Youngstrom and Strowbridge, 2015). To our knowledge, there have been no studies on GC activity in awake behaving animals. Here, we used fiber photometry a sensitive but easy method of detecting changes in fluorescence in a population of cells to monitor GC activity in mice engaging in a go/no go task. Although the population recorded may contain a small fraction of short axon cells, another interneuron subtype in the granule cell layer (Eyre et al., 2008; Nagayama et al., 2014), the vast majority of GABAergic neurons in this layer are GCs. Robust and reliable responses were recorded in all mice tested and the calcium signals changed when different odors were presented while remaining stable across different trials of the same odor, indicating that this method works well for characterizing odor-evoked neural activity in GCs.

Studies from electrophysiological recording, two-photon imaging, and fiber photometry recording consistently demonstrate both excitatory and inhibitory responses to passive odor exposure in M/T cells (Yamada et al., 2017; Wang et al., 2019; Liu et al., 2020). By contrast, we observed only excitatory responses to passive odor exposure in GCs, consistent with our previous study (Sun et al., 2019). Indeed, excitatory odor responses have also been found in previous studies using two-photon calcium imaging and extracellular recording (Kato et al., 2012; Cazakoff et al., 2014). There are mainly two types of glutamatergic inputs onto GCs in the OB: the dendrodendritic input from mitral cells in the external plexiform layer and the axodendritic input from the olfactory cortex in the granule cell layer (Balu et al., 2007; Pressler and Strowbridge, 2017, 2019). Both of these two inputs may contribute to the odor responses in GCs. The optical fibers were embedded in the granule cell layer and GCs show several types of dendritic spikes that might not necessarily reach the soma (Zelles et al., 2006; Lin et al., 2007). In addition, a recent study has revealed that the unitary dendrodendritic input is relatively weak with highly variable release probability but cortical input to GCs is more powerful and less variable (Pressler and Strowbridge, 2017). Thus, the excitatory responses recorded in this study mainly reflect the cortical feedback input from the olfactory cortex onto GCs. Our previous studies have shown that odors always evoke excitatory responses in the pyramidal neurons of the piriform cortex (Zhou et al., 2017; Wang et al., 2020). Given that granule cells in the OB receive extensive glutamatergic feedback from the olfactory cortex (Boyd et al., 2012), the excitatory response in GCs mainly derive from the olfactory cortex. In future studies, more direct evidence could be provided by recordings focused on more subtle processing in the external plexiform layer of the OB in behaving animals. Although previous studies have shown a lack of learning-related plasticity in the piriform cortex (Zinyuk et al., 2001; Wang et al., 2019), whether the cortical inputs to GCs display such plasticity is unknown. Indeed, long-term plasticity has been induced in the cortical feedback inputs to GCs (Gao and Strowbridge, 2009; Cauthron and Stripling, 2014) and an olfactory circuitry model suggests that changes in the weight of top-down feedback contribute to pattern separation (Chen and Padmanabhan, 2020). Therefore, changes in the weight of cortical inputs may regulate the plasticity in GCs during odor discrimination learning.

Since the separation of odor responses in OSN inputs remains stable during perceptual learning (Chu et al., 2017), the circuits within and/or beyond the OB must be responsible for the increase in pattern separation with learning in M/T cells. Here we find that odor responses in GCs also display improved separation, suggesting that granule cells may mediate the pattern separation in M/T cells. There are several lines of evidence in support of this hypothesis. (1) Disruption of GABAergic inhibition onto M/T cells impairs pattern separation in M/T cells and odor discrimination (Godde et al., 2016). Furthermore, bidirectional manipulation of GC activity affected pattern separation in M/T cells and odor discrimination performance (Gschwend et al., 2015; Nunes and Kuner, 2015). (2) Cortical feedback enhances pattern separation in mitral cells through inhibitory circuits (Otazu et al., 2015). Since granule cells are the main target of the feedback fibers, they may contribute to the cortical regulation of mitral cell pattern separation. (3) Indeed, it was recently shown that cortical feedback via granule cells in the OB could account for the learning-related pattern separation in mitral cells (Yamada et al., 2017). These studies combined with our findings suggest that GCs in the OB likely mediate the pattern separation in M/T cells. This hypothesis is further supported by a recent study showing that odor-induced changes in the power of local field potential oscillations in the OB also display learning-related separation (Ramirez-Gordillo et al., 2018). A previous study has shown that GC-specific silencing does not alter the firing rate of mitral cell (Fukunaga et al., 2014). In the future, it would be important to examine whether and how GCs mediate the pattern separation in M/T cells.

Furthermore, neuromodulatory inputs to the OB, including noradrenergic, serotonergic, and cholinergic fibers, have been demonstrated to shape the responses of mitral cells to odor and play an important role in olfactory-related behavior (Doucette and Restrepo, 2008; Escanilla et al., 2010; Ma and Luo, 2012; Nunez-Parra et al., 2013; Kapoor et al., 2016). Optogenetic silencing of noradrenergic axons in the OB disrupts learning-related separation in OB oscillations (Ramirez-Gordillo et al., 2018). Raphe activation, and presumed subsequent increases in endogenous serotonin release, leads to increased pattern separation in mitral cell odor codes (Kapoor et al., 2016). Future studies are needed to determine whether and how neuromodulation regulates the improved pattern separation observed in the OB during olfactory discrimination.

In summary, the present study provides direct evidence that odor responses of granule cells in the OB show improved separation during odor discrimination learning, suggesting task-dependent plasticity in the response of granule cells to odors. This finding is important for understanding the function of different cell types in the OB and how the OB processes odor information in the ever-changing real environment.

## Data Availability Statement

The raw data supporting the conclusions of this article will be made available by the authors, without undue reservation, to any qualified researcher.

## Ethics Statement

The animal study was reviewed and approved by the Xuzhou Medical University Institutional Animal Care and Use Committee.

## Author Contributions

DW and AL designed the research and wrote the manuscript. YaC, DW, XL, and ZY performed the research. DW, YaC, YiC, and PL analyzed the data. All authors contributed to the article and approved the submitted version.

## Conflict of Interest

The authors declare that the research was conducted in the absence of any commercial or financial relationships that could be construed as a potential conflict of interest.
